# Spatial Pattern and Dynamic Change of Vegetation Greenness From 2001 to 2020 in Tibet, China

**DOI:** 10.3389/fpls.2022.892625

**Published:** 2022-04-25

**Authors:** Fugen Jiang, Muli Deng, Yi Long, Hua Sun

**Affiliations:** ^1^Research Center of Forestry Remote Sensing and Information Engineering, Central South University of Forestry and Technology, Changsha, China; ^2^Key Laboratory of Forestry Remote Sensing Based Big Data and Ecological Security for Hunan Province, Changsha, China; ^3^Key Laboratory of State Forestry Administration on Forest Resources Management and Monitoring in Southern Area, Changsha, China

**Keywords:** vegetation greenness, ecosystem monitoring, spatial–temporal analysis, Google earth engine, Hurst exponent

## Abstract

Due to the cold climate and dramatically undulating altitude, the identification of dynamic vegetation trends and main drivers is essential to maintain the ecological balance in Tibet. The normalized difference vegetation index (NDVI), as the most commonly used greenness index, can effectively evaluate vegetation health and spatial patterns. MODIS-NDVI (Moderate-resolution Imaging Spectroradiometer-NDVI) data for Tibet from 2001 to 2020 were obtained and preprocessed on the Google Earth Engine (GEE) cloud platform. The Theil–Sen median method and Mann–Kendall test method were employed to investigate dynamic NDVI changes, and the Hurst exponent was used to predict future vegetation trends. In addition, the main drivers of NDVI changes were analyzed. The results indicated that (1) the vegetation NDVI in Tibet significantly increased from 2001 to 2020, and the annual average NDVI value fluctuated between 0.31 and 0.34 at an increase rate of 0.0007 year^−1^; (2) the vegetation improvement area accounted for the largest share of the study area at 56.6%, followed by stable unchanged and degraded areas, with proportions of 27.5 and 15.9%, respectively. The overall variation coefficient of the NDVI in Tibet was low, with a mean value of 0.13; (3) The mean value of the Hurst exponent was 0.53, and the area of continuously improving regions accounted for 41.2% of the study area, indicating that the vegetation change trend was continuous in most areas; (4) The NDVI in Tibet indicated a high degree of spatial agglomeration. However, there existed obvious differences in the spatial distribution of NDVI aggregation areas, and the aggregation types mainly included the high-high and low-low types; and (5) Precipitation and population growth significantly contributed to vegetation cover improvement in western Tibet. In addition, the use of the GEE to obtain remote sensing data combined with time-series data analysis provides the potential to quickly obtain large-scale vegetation change trends.

## Introduction

As a link between the atmosphere, soil, and water bodies, vegetation constitutes an indispensable component of terrestrial ecosystems and plays an important role in the material cycle and energy flow (Ni, 2001; Yan et al., 2020). Environmental and climate problems such as soil erosion, soil desertification, and the greenhouse effect caused by vegetation destruction cannot be ignored (Fattet et al., 2011; Dong et al., 2021). It is crucial to monitor and predict vegetation change trends and identify associated drivers (Fensholt et al., 2009).

Tibet is the main body of the Tibetan Plateau and the birthplace of the Yangtze and Yarlung Tsangpo rivers, whose ecological changes affect the climate of East Asia and even the world (Luo et al., 2018; Yi et al., 2018). However, under global warming and enhanced human activities, environmental problems such as desertification are becoming increasingly serious (Wang et al., 2016). Due to the complex influences of harsh climatic and geographical conditions, the vegetation ecosystem in Tibet is fragile and sensitive, and there exists notable spatial heterogeneity in the relationship between vegetation and climate and human activities (Immerzeel et al., 2008). Monitoring Tibetan vegetation trends and identifying its response to climate change and other factors can deepen the understanding of vegetation change mechanisms on the Tibetan Plateau, which is essential for the conservation of vegetation ecosystems and environmental restoration in alpine regions (Chen et al., 2020).

The methods for vegetation surveys in alpine regions mainly include field surveys and remote sensing detection. Field surveys are highly accurate; however, the harsh environment and vastness of the area make manual surveys extremely difficult, and real-time vegetation renewal across the whole area is almost unattainable (Li et al., 2014). Remote sensing technology, with its fast, real-time, and wide coverage, provides a new and convenient way to monitor terrestrial ecosystems and is widely used in areas such as vegetation growth management and remote sensing for land cover change monitoring (Zhan et al., 2002; Jiang et al., 2021). The use of remote sensing data sources to construct vegetation indices sensitive to vegetation growth has become a major method to monitor and assess regional vegetation environments. The normalized difference vegetation index (NDVI), as an index representing vegetation greenness, can visually reflect the vegetation growth status and distribution density and is an important index for vegetation change monitoring and climate response research (Rouse et al., 1974; Yuan and Bauer, 2007). In recent years, the use of remote sensing data to extract NDVI time series for vegetation growth monitoring has become one of the main ways to evaluate vegetation ecosystems in large regions (Jiang et al., 2015; Zou et al., 2020). However, the acquisition of real-time vegetation NDVI data in alpine regions is always limited due to the cloud volume, data availability, and computational efficiency. In addition, existing studies on vegetation dynamics in Tibet or the Qinghai-Tibet Plateau involving remote sensing require massive data download and preprocessing procedures, which represents a very high workload and an extremely time-consuming endeavor, with limited applications in efficient large-scale vegetation monitoring. The Google Earth Engine (GEE) is an online cloud platform for data processing that can quickly acquire and batch process massive remote sensing data (Dong et al., 2016; Gorelick et al., 2017). Currently, the GEE has been successfully used to acquire remote sensing images such as Landsat or Sentinel data for mangrove monitoring, land cover change determination, and deforestation detection (Tamiminia et al., 2020; Samanta et al., 2021). In addition, MODIS data that can provide periodic surface information is also provided in GEE. As an evaluation index of vegetation greenness, MODIS-NDVI has the potential to quickly identify and monitor large-scale vegetation (Jepsen et al., 2009). However, the efficiency and effectiveness of the GEE in the acquisition of time-series data of large areas for vegetation greenness monitoring in alpine and high-altitude regions require further validation.

Time-series vegetation indices for vegetation change evaluation have been widely employed (Jiang et al., 2015; Zou et al., 2020). In Wang and Han (2012), based on meteorological data and SPOT vegetation NDVI data from 1999 to 2008, linear correlation analysis was performed to analyze the spatial and temporal variation patterns of the vegetation cover across the Tibetan Plateau. The results indicated that the annual NDVI exhibited a significant increasing trend and that the ecological environment of the Tibetan Plateau was developing along a favorable direction under the influence of climate change. Ding et al. (2015) successfully obtained the start of the growing season (SGS) on the Tibetan Plateau from 1982 to 2012 based on normalized difference vegetation index (NDVI) data obtained from the GIMSS and SPOT. Zou et al. (2020) calculated the spatial and temporal trends of vegetation indices and surface temperature on the Tibetan Plateau and explored the relationship between vegetation and surface temperature changes and climatic factors. The results demonstrated that the vegetation cover on the Tibetan Plateau generally followed an increasing trend and significant spatial and temporal heterogeneity levels from 2001 to 2012. However, these studies mainly focused on national scales or the entire Tibetan Plateau region. This could ignore the local distribution characteristics of Tibetan vegetation due to spatial heterogeneity and the specificity of altitude and climate. In addition, the validity and timeliness of the research cycles selected in these studies have progressively become inadequate.

Trends in vegetation dynamics can reflect the direction of vegetation change during the study period, and possible trends can be predicted, which can guide the implementation of specific measures to manage future vegetation changes. The Hurst exponent, which can reflect the autocorrelation of time series and hidden long-term series trends, has been widely used in hydrological, meteorological, and environmental research. Studies have used the Hurst exponent to predict future vegetation dynamics. Peng et al. (2012) used the Hurst exponent method to predict future vegetation changes on the Tibetan Plateau based on an AVHRR GIMMS-NDVI dataset from 1982 to 2003, and the results indicated that the obtained future vegetation change trends were consistent across most of the Tibetan Plateau. Notably, Chen et al. (2020) used the Hurst exponent method to demonstrate that the Tibetan Plateau occurs at a high risk of vegetation degradation. However, as one of the main bodies of the Qinghai-Tibetan Plateau, the prediction of future vegetation change based on historical trends in Tibet has not been reported.

In this study, MODIS-NDVI data were obtained based on the GEE platform to reveal the latest trends of vegetation change in Tibet from 2001 to 2020 and to quantify the contribution of climate change and human activities. To reduce the influence of outliers, a more robust Theil–Sen median method and Mann–Kendall test were employed to evaluate the spatial patterns and trends of the NDVI. The Hurst exponent was established to predict the future trend of vegetation. In addition, the spatial autocorrelation of the NDVI in Tibet was examined to provide a scientific basis for ecological environment construction in the Tibetan Plateau region and other alpine regions.

## Materials and Methods

### Study Area

**Figure 1:**
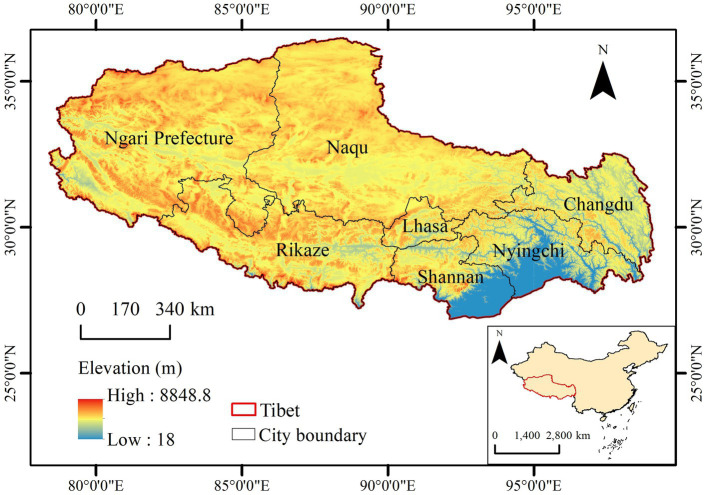
Location and altitude distribution in the study area.

Tibet is located on the Qinghai-Tibet Plateau (78°25′–99°06′E, 26°50′–36°53′N) (Figure 1) in southwestern China. Due to the altitude and latitude, the climate difference between Southern and Northern Tibet is obvious. Southern Tibet is mild and rainy, with an annual average temperature of 8°C. Northern Tibet exhibits a typical continental climate, with an annual average temperature below 0°C and a freezing period longer than 6 months. With a total area of 1,228,400 km^2^ and an average altitude exceeding 4 km, the distribution of water and heat resources is uneven, and the ecosystem is relatively fragile. The vegetation types mainly include forests, meadows, grasslands, deserts, and alpine vegetation. The area of natural pastures is 83 million hectares, accounting for 67% of the land area of the whole region, and the forest coverage reaches 6.32 million hectares. And the main tree species include spruce (*Picea asperata* Mast), fir (*Abies fabri* (Mast.) Craib), and larch (*Larix ologensis*).

### Data Sources

NDVI data were provided by the National Aeronautics and Space Administration (NASA) MODIS Terra (MOD13Q1) satellite and acquired from the GEE cloud platform. The acquired MOD13A2 data encompassed 16-day vegetation index products, which have been verified to effectively reflect the vegetation growth status. MOD13A2 began providing vegetation index data with a spatial resolution of 1 km in February 2000, and low-cloud and low-view NDVI values were selected from all acquisitions over 16 days to ensure the best available pixel values (Fensholt et al., 2009). NDVI images from 2001 to 2020 were obtained and preprocessed *via* reprojection, splicing, and clipping. To eliminate the influence of clouds, the maximum value compositing (MVC) method was applied to all pixels to obtain the best annual grid data over 20 years (Leeuwen et al., 1999).

**Figure 2:**
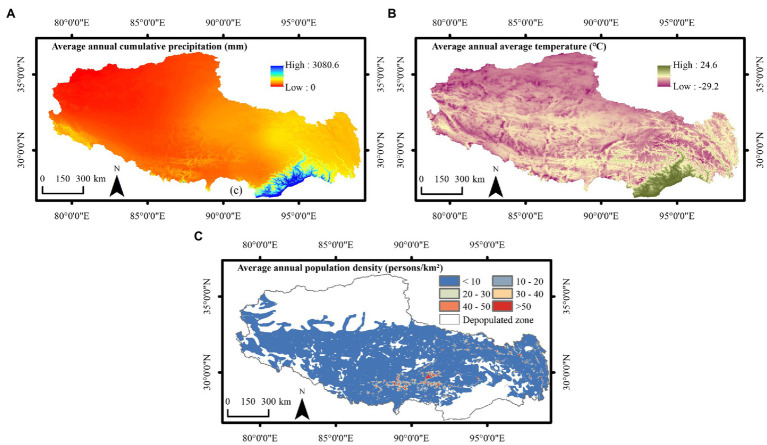
Spatial pattern of the average values of **(A)** the annual cumulative precipitation, **(B)** annual average temperature, and **(C)** annual population density in Tibet from 2001 to 2020.

To identify the drivers of NDVI change, climate change and human activity factors were selected for comparison and analysis. The drivers considered in this study included the annual cumulative precipitation, annual average temperature, annual population density data, and nighttime light data. Precipitation and temperature data were obtained from the Resource and Environmental Science and Data Center of the Chinese Academy of Sciences,^1^ population density data were obtained from WorldPop,^2^ and nighttime light data were obtained from the NPP-VIIRS data website.^3^ Due to the limitation of the time-series length of nighttime light data, data from 2013 to 2020 were selected. The resolution of all data was resampled to match the 1 km spatial resolution of the NDVI data to ensure consistency between the different data sources (Figure 2). In addition, major meteorological and geological disasters in Tibet and important policies on vegetation were obtained from the Statistical Yearbook of Tibet (Statistical Bureau of Tibet, 2015, 2016).

### Methods

#### Coefficient of Variation

The coefficient of variation (*Cv*) can suitably reflect the time-based difference and change degree of spatial data and can be used to evaluate the stability of time-series data (Weber et al., 2004). The larger the *Cv* value is, the more discrete the distribution of the NDVI values and the more drastic the vegetation changes, while the smaller the *Cv* value is, the more concentrated the distribution of the NDVI values and the more stable the vegetation. Coefficient of variation values of the NDVI was calculated by the pixel to analyze the NDVI difference in Tibet and its stability over 20 years. *Cv* can be calculated with Equation 1.


(1) $$C_{v}=\frac{\sqrt{\frac{1}{n}\sum_{i=1}^{n}{\left(NDVI_{i}-\bar{\mathrm{NDVI}}\right)}^{2}}}{\bar{\mathrm{NDVI}}}$$

where *C_v_* is the coefficient of variation, $NDVI_{i}$ is the NDVI value in year *i*, and $\bar{\mathrm{NDVI}}$ is the average NDVI value from 2001 to 2020.

#### Trend Analysis

The Theil–Sen median method is a robust nonparametric approach for trend calculation, which is often used in combination with the Mann–Kendall test to evaluate the trend and significance of time-series data (Fernandes and Leblanc, 2005; Kisi and Ay, 2014). This method is insensitive to measurement errors and outlier data, which can reduce the influence of outliers on the results and has been widely used in trend analysis of long time-series data (Jiang et al., 2015). The equation to calculate *β* in the Theil–Sen median method is as follows:


(2) $$\beta =\mathrm{median}\left(\frac{NDVI_{j}-NDVI_{i}}{j-i}\right),2001\leq i<j\leq 2020$$

where $NDVI_{j}$ and $NDVI_{i}$ are the NDVI values in years *j* and *i*, respectively. In this study, 2020 ≥ *j* ≥ *i* ≥ 2001. Additionally, when β is greater than 0, the vegetation NDVI exhibits an increasing trend; when β is less than 0, the NDVI exhibits a decreasing trend; and when β is equal to 0, the NDVI remains stable and unchanged. The vegetation NDVI trend results can be classified into five classes: significant degradation, slight degradation, stable unchanged, slight improvement, and significant improvement.

#### Hurst Exponent

The Hurst exponent method based on rescaled interval (R/S) analysis is a time-series analysis method based on fractal theory and exhibits wide applications in the fields of climate change and population migration (Peng et al., 2012). R/S analysis can measure how the fluctuation range of a given time series varies with the time span, which can be used to predict the future trend of vegetation (Jiang et al., 2015; Li et al., 2021).

The main principle of R/S analysis is the development of a time series that defines an average series and {NDVI(*t*), *t* = 1, 2,····n}, for any positive integer *τ* ≥ 1. The calculation procedures are as follows:

Defined mean sequence:


(3) $${\bar{\mathrm{NDVI}}}_{\left(\tau \right)}=\frac{1}{\tau }\overset{\tau }{\underset{t=1}{\sum }}NDVI_{\left(\tau \right)},\tau =1,2,3,\ldots ,n$$

Cumulative deviation:


(4) $$X\left(t,\tau \right)=\overset{t}{\underset{t=1}{\sum }}\left(NDVI_{\left(t\right)}-{\bar{\mathrm{NDVI}}}_{\left(\tau \right)}\right),1\leq t\leq \tau$$

Range:


(5) $$R_{\left(\tau \right)}=\max_{1\leq t\leq \tau }X\left(t,\tau \right)-\min_{1\leq t\leq \tau }X\left(t,\tau \right),\tau =1,2,3,\ldots ,n$$

Standard deviation:


(6) $$S_{\left(\tau \right)}={\left[\frac{1}{\tau }\overset{\tau }{\underset{t=1}{\sum }}{\left(NDVI_{\left(t\right)}-NDVI_{\left(\tau \right)}\right)}^{2}\right]}^{\frac{1}{2}},\tau =1,2,3,\ldots ,n$$

Hurst exponent:


(7) $$\frac{R_{\left(\tau \right)}}{S_{\left(\tau \right)}}={\left(c\tau \right)}^{H}$$

where *H* is the Hurst exponent, which is calculated *via* the least square method. For 0.5 < *H* ≤ 1, this indicates that vegetation change exhibits persistence, and the future change trend is consistent with past change trends, and the larger *H* is, the stronger the persistence. For *H* = 0.5, the vegetation change exhibits randomness, and the future change trend cannot be determined. For 0 ≤ *H* < 0.5, this suggests that the determined vegetation change exhibits inverse persistence, and the future change trend is the opposite to past change trends. In addition, the NDVI change trend was coupled with the Hurst exponent to obtain the persistence in the NDVI change trend. The definition of the trend is ruled as shown in Table 1.

**Table 1:** The rule of definition of the NDVI change trend.

Standard of classification	Hurst exponent & trend
0.5 < *H* ≤ 1 and *β* < −0.0005, |Z| > 1.96	Sustainability & Significant degradation
0.5 < *H* ≤ 1 and *β* < −0.0005, |Z| ≤ 1.96	Sustainability & Slight degradation
0.5 < *H* ≤ 1 and −0.0005 ≤ *β* ≤ 0.0005	Sustainability & Stable unchanged
0.5 < *H* ≤ 1 and *β* > 0.0005, |Z| ≤ 1.96	Sustainability & Slight improvement
0.5 < *H* ≤ 1 and *β* > 0.0005, |Z| > 1.96	Sustainability & Significant improvement
0 ≤ *H* ≤ 0.5	Uncertainty future trend

#### Correlation Analysis

Pearson correlation coefficient values were separately calculated in R software using climate factors and human activity data contemporaneous with the above NDVI time series to reveal the main drivers of NDVI changes (Jiang et al., 2015; Sun et al., 2019). Pearson’s correlation can be expressed by *R*, as calculated with Equation (8). Positive or negative values indicate whether the drivers are positively or negatively correlated, respectively, with the NDVI. Larger absolute values indicate stronger correlations.


(8) $$R_{xy}=\frac{\sum_{i=1}^{n}\left[\left(x_{ij}-\bar{x_{j}}\right)\left(y_{ij}-\bar{y_{j}}\right)\right]}{\sqrt{\sum_{i=1}^{n}{\left(x_{ij}-\bar{x_{j}}\right)}^{2}\sum_{i=1}^{n}{\left(y_{ij}-\bar{y_{j}}\right)}^{2}}}$$

where *n* is 20, $x_{ij}$ and $y_{ij}$ denote the individual values of the drivers and NDVI, respectively, in the *i*th year, while $\bar{x_{j}}$ and $\bar{y_{j}}$ are the mean values of the drivers and NDVI, respectively, over 20 years.

## Results

### Spatial Pattern of the Vegetation NDVI

**Figure 3:**
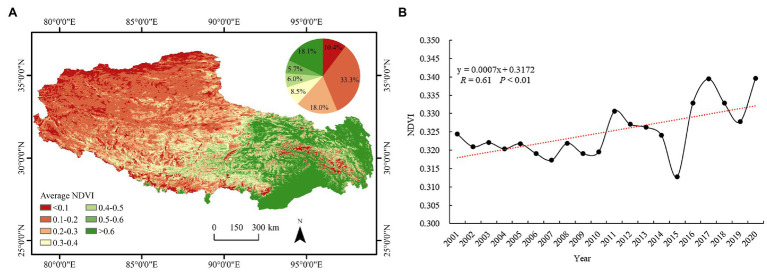
**(A)** Spatial pattern of the average values and **(B)** interannual variation in the NDVI in Tibet from 2001 to 2020.

Figure 3A shows the spatial distribution pattern of the average NDVI in Tibet from 2011 to 2020. The NDVI value in Tibet approximately decreased from southeast to northwest. Northwestern Tibet mainly comprises bare land and snowy areas with dry and cold climatic conditions and poor vegetation ecological conditions, resulting in low NDVI values. The southeastern region mainly includes a valley plain with a low altitude, belonging to semihumid and humid climate areas, with suitable hydrothermal conditions and an excellent ecological environment. This region is the main distribution area of crops and woodlands in Tibet, so the NDVI value is high. The statistical results of the average NDVI values in Tibet over the past 20 years indicated that the nonvegetated area with an NDVI value below 0.1 accounted for 10.4% of the total plateau area and the area with a low NDVI value (0.1–0.4) accounted for 59.8% of the total plateau area. The area with an NDVI value ranging from 0.5 to 0.6 accounted for 5.7% of the total plateau area, and the area with an NDVI value above 0.6 accounted for 18.1% of the total plateau area.

To clarify the state of the vegetation cover in Tibet and the characteristics of vegetation NDVI changes over time, the interannual NDVI change trend was mapped (Figure 3B). The annual average NDVI in Tibet increased and fluctuated between 0.31 and 0.34 at a rate of 0.0007 year^−1^, among which the vegetation NDVI exhibited a slowly fluctuating decreasing trend from 2001 to 2010. However, the fluctuation in the NDVI from 2010 to 2020 was more drastic and revealed an overall increasing trend, indicating that the vegetation cover conditions gradually improved. In 2015, the annual cumulative precipitation in Tibet significantly decreased, and extreme disaster weather events, such as severe snowfall, drought, and hailstorms, occurred in different areas, resulting in the lowest vegetation NDVI values in 20 years. In response, the Chinese government adopted a series of activities and policies including the construction of protective forest system, sand control and management, and return of cultivated land to forest to realize revegetation from 2015 to 2016, resulting in a significant increase in vegetation cover.

**Figure 4:**
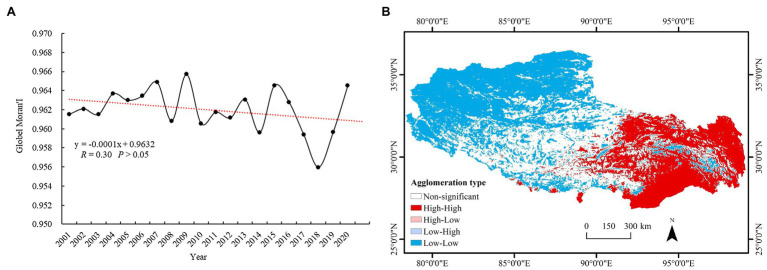
**(A)** Global Moran’s index variation in the NDVI and **(B)** spatial pattern of local Moran’s index in Tibet from 2001 to 2020.

To detect the aggregation features and local distribution pattern of the NDVI in Tibet, global Moran’s index and local Moran’s index values were calculated. From 2001 to 2020, global Moran’s index of the NDVI in Tibet fluctuated between 0.956 and 0.966 (*p* < 0.01), indicating that the NDVI exhibits high spatial agglomeration (Figure 4A). In addition, global Moran’s index fluctuated sharply from 2001 to 2015, exhibiting a downward trend, indicating that the spatial agglomeration degree gradually decreased. Figure 4B shows that there existed obvious differences in the spatial distribution of the vegetation NDVI aggregation areas in Tibet, and the aggregation types mainly included the high-high and low-low types. Low-low type areas were mainly distributed in the west and north, and the associated patches were large. High-high type areas were mainly distributed in the east and south. The areas without significant aggregation were mainly concentrated in the central region, and the patches were relatively discontinuous. There occurred few low-high and high-low aggregation areas. The NDVI values in the western and northern regions of Tibet were generally low, while those in the eastern region were generally high (Figure 4B).

### Stability of Vegetation NDVI Changes

**Figure 5:**
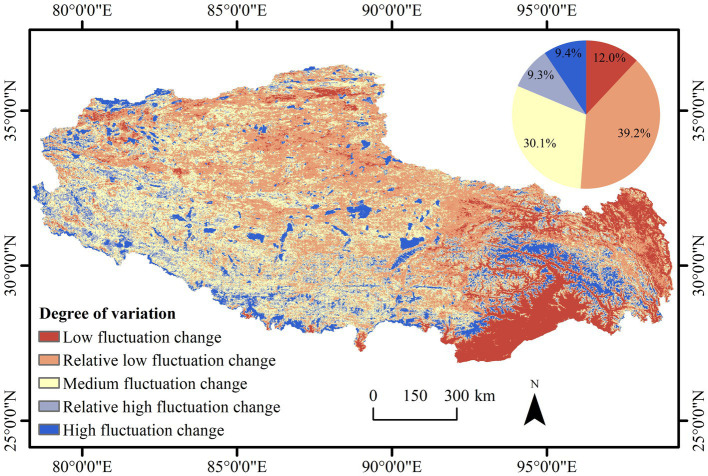
Spatial distribution of the coefficient of variation of the NDVI from 2001 to 2020 in Tibet.

The mean value of the coefficient of variation of the vegetation NDVI in the study area was 0.13, and the area exhibiting relatively high and high-fluctuation changes jointly accounted for 18.7% of the total area (Figure 5). The order of the areas considering each degree of variation was relatively low fluctuation change > medium fluctuation change > low fluctuation change > relatively high fluctuation change > high-fluctuation change (Table 2). Areas with a low fluctuation change in the NDVI mainly occurred in the southeast and northeast, where the climate is warm and humid, the vegetation types are abundant, the vegetation growth conditions are superior, and the vegetation NDVI was generally high and stable in the time series. Areas with a high-fluctuation change were scattered in the west, south-central, and east. The western part belongs to the highland area, where the ecosystem is very fragile and vulnerable to the natural environment. The south-central region exhibits a high population density and urban development level, leading to drastic vegetation changes, which in turn is reflected in the high fluctuation in the vegetation NDVI. The high-fluctuation area in the east is mainly the water area.

**Table 2:** Coefficient of variation statistics of the NDVI from 2001 to 2020 in Tibet.

Coefficient of variation	Degree of variation	Proportion/%
*C_v_* ≤ 0.05	Low fluctuation change	12.0
0.05 < *C_v_* ≤ 0.10	Relatively low fluctuation change	39.2
0.10 < *C_v_* ≤ 0.15	Medium fluctuation change	30.1
0.15 < *C_v_* ≤ 0.20	Relatively high-fluctuation change	9.3
*C_v_* ≥ 0.20	High-fluctuation change	9.4

### Trend Changes of the Vegetation NDVI

**Figure 6:**
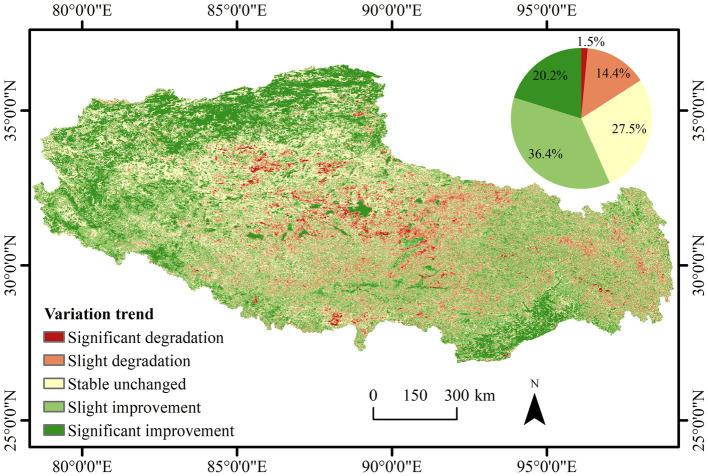
Spatial pattern of the NDVI change trends from 2001 to 2020 in Tibet.

The NDVI trends of Tibetan vegetation revealed significant regional differences in the spatial distribution. The stable areas were mainly located in the western part of the study area, while the slightly degraded and significantly degraded areas were mainly located in the central and northern parts, respectively, of the study area. The NDVI trends in the central and eastern parts of the study area were different and more notably fragmented. The areas with an improved vegetation cover in Tibet over the past 20 years were larger than the areas with a degraded vegetation cover. The area with an improved vegetation cover accounted for 56.6% of the total area of the region, the area with a stable and unchanged vegetation cover accounted for 27.5% of the total area, and the area with a degraded vegetation cover accounted for only 15.9% of the total area (Figure 6).

### Sustainability of Vegetation NDVI Variations

**Figure 7:**
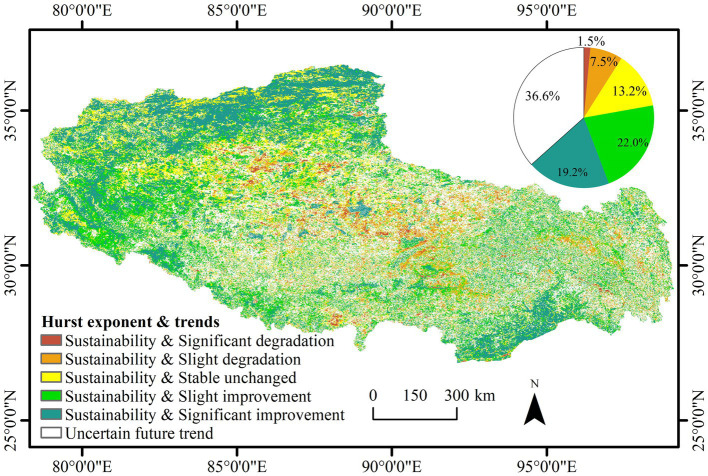
Spatial distribution of the NDVI trends based on the Hurst exponent.

The mean Hurst exponent of the NDVI in Tibet reached 0.53. Areas with a Hurst exponent smaller than 0.5 accounted for 36.6% of the total study area, and the percentage of areas with a Hurst exponent larger than 0.5 was 63.4%, indicating a strong positive persistence of the vegetation NDVI in general. The results of the vegetation NDVI trends were superimposed and coupled with the Hurst exponent to obtain vegetation NDVI trends and their persistence (Figure 7). The results could be classified as sustainability and significant degradation, sustainability and slight degradation, sustainability and stable unchanged, sustainability and slight improvement, sustainability and significant improvement, and uncertain future trends.

The area of continuously improving regions account for 41.2% of the total area, mainly distributed in the northwest and southeast; the area of continuously stable and unchanged regions accounted for 13.2% of the total area, mainly distributed in the central and western regions; the area of continuously degraded regions accounted for 9.0% of the total area, scattered in the central region; and the area of regions with uncertain future change trends accounted for 36.6% of the total area, mainly distributed in small parts of the eastern and central regions.

### Correlation Analysis Between Drivers and NDVI Changes

**Figure 8:**
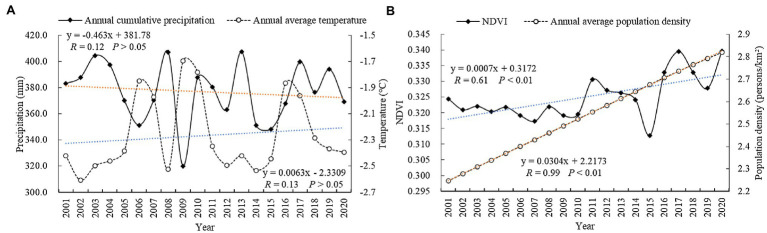
Variations in the **(A)** annual cumulative precipitation, annual average temperature, **(B)** NDVI, and annual population density data in Tibet from 1997 to 2017.

Over the past 20 years, the average annual cumulative precipitation in Tibet reached approximately 396.91 mm, and the annual cumulative precipitation fluctuated within the range from 319.96 to 407.43 mm, at a rate of 0.463 mm year^−1^. The annual average temperature was approximately −2.27°C, fluctuating within the range from approximately −1.70°C to −2.61°C, at a rate of 0.0063°C year^−1^ (Figure 8A). The trend of the annual cumulative precipitation was the opposite to that of the annual average temperature, and the trend of climate change in Tibet indicated warm and dry conditions. Moreover, the changes in both the annual average population density and NDVI in Tibet over the last 20 years exhibited increasing trends, and the annual average population density increased year by year at a rate of 0.0304 persons year^−1^. The minimum value of the population density was 2.25 persons/km^2^, and the maximum value was 2.81 persons/km^2^ (Figure 8B).

The correlations between the annual cumulative precipitation and annual average temperature and the average annual NDVI were not significant. However, the average annual population density exhibited a significant positive correlation with the average annual NDVI with a correlation coefficient of 0.61 (*p* < 0.01). These results indicated that precipitation and temperature imposed no significant effect on vegetation cover recovery and that human activities were the main drivers of NDVI changes in Tibet as a whole.

**Figure 9:**
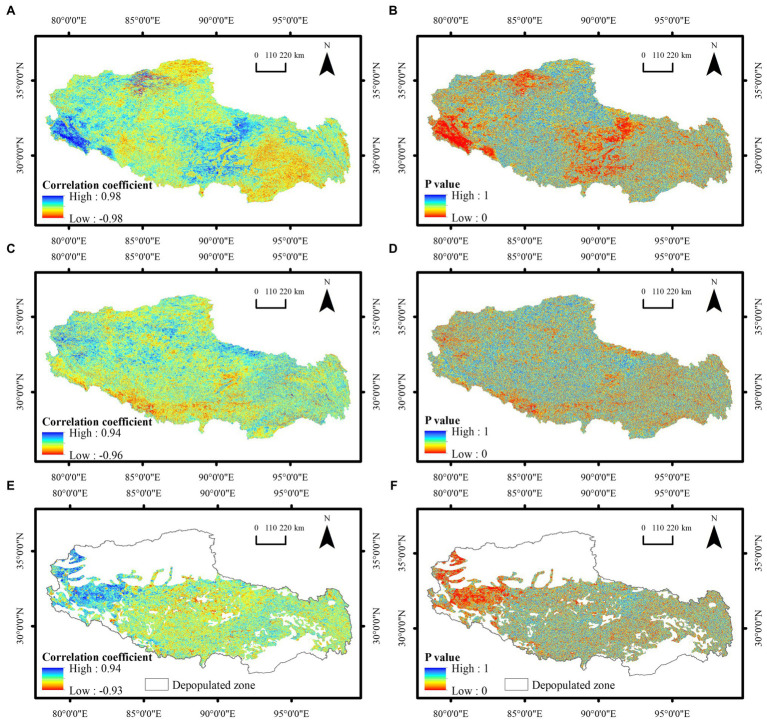
Spatial distribution of the correlation coefficient values and significance between the NDVI and **(A)**, **(B)** annual cumulative precipitation, **(C)**, **(D)** annual average temperature, and **(E)**, **(F)** annual population density in Tibet from 2001 to 2020.

To capture local information on the drivers of NDVI changes in Tibet in more detail and precision, the correlation coefficient values between the NDVI and the annual cumulative precipitation, annual average temperature, and population density were calculated pixel by pixel, and spatial distributions were obtained (Figure 9). The correlation coefficient values between the NDVI and annual cumulative precipitation in Tibet ranged from −0.98 to 0.98, with the areas with a significant negative correlation accounting for approximately 1.7% of the total area, and those with a significant positive correlation accounting for approximately 8.2% of the total area, mainly in the western and central regions. The correlation coefficient values between the NDVI and annual average temperature ranged from −0.96 to 0.94, with the areas with significant positive and negative correlations accounting for approximately 2.8% of the total area, and those with significant negative correlations mainly occurring in the western and central regions. In addition, the correlation coefficient values between the NDVI and population density varied between −0.93 and 0.94, with 1.4% of the regions exhibiting a significant negative correlation and 5.0% of the regions attaining a significant positive correlation, which were mainly distributed in the western region.

**Figure 10:**
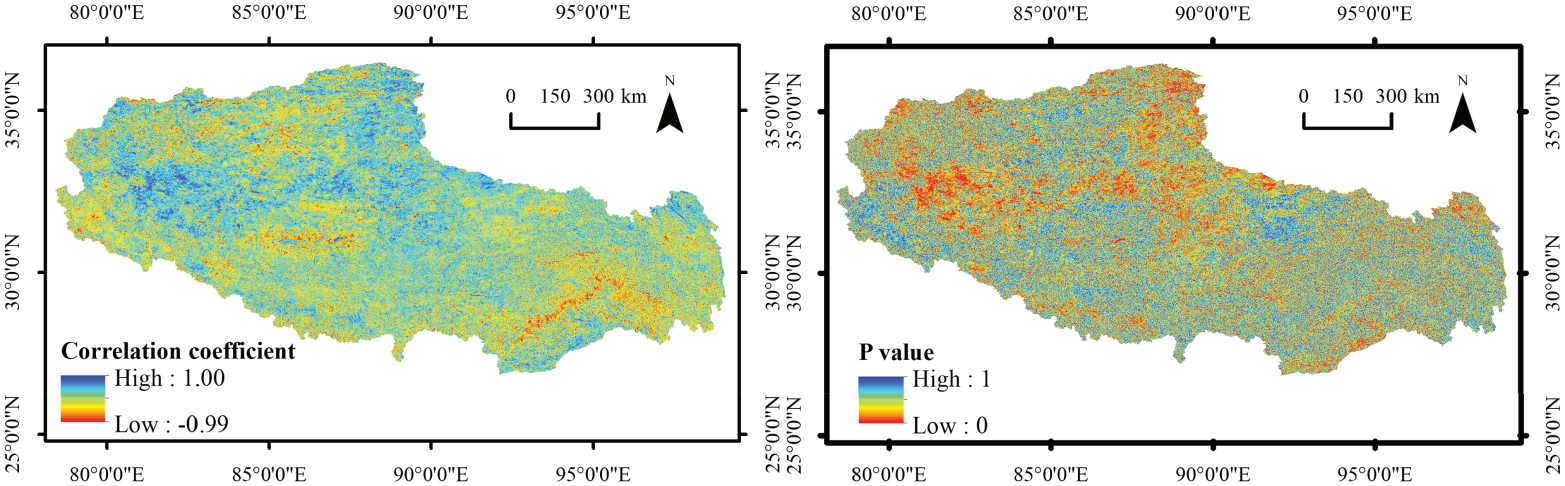
Spatial distribution of correlation coefficients and significance between NDVI and nighttime light data in Tibet from 2013 to 2020.

To further identify the impact of human activities on NDVI changes in Tibet, nighttime light data were used for correlation analysis. The results indicated that the annual average nighttime light intensity in Tibet fluctuated and increased with the annual NDVI. There existed a significant correlation between the NDVI and nighttime light data in most parts of western Tibet, which further verified that human activities represented the main factor promoting vegetation activities (Figure 10).

## Discussion

### Vegetation NDVI Change Trend and Drivers

Climate change and human activities have been verified as the main factors causing vegetation change (Zhang et al., 2018; Zou et al., 2020). Vegetation is particularly sensitive to climate change during its growth period, especially in high-altitude areas with an extremely fragile ecology (Zhou et al., 2004; Xu et al., 2017). The results in our study demonstrated that the NDVI in Tibet has slightly increased over the last 20 years, similar to the conclusion obtained by Zhang et al. (2018) regarding the overall vegetation on the Tibetan Plateau. However, vegetation degradation in central and northern Tibet cannot be ignored. Global warming and drought are the main causes of vegetation degradation in northern regions, while human activities significantly impact vegetation recovery in central regions. Precipitation is not the dominant climatic factor of the increase in vegetation greenness in Tibet, but the fragile alpine grassland ecosystem is vulnerable to climate events, which leads to drastic changes in biodiversity and affects vegetation greening. In addition, Liu et al. (2021) demonstrated that across the whole Qinghai-Tibet Plateau, climate warming, humidity, and livestock control contribute to the significant vegetation restoration. With accelerated urbanization, destruction of vegetation can occur in some areas, but measures such as ecological reforestation projects and natural forest protection projects in China have been verified to positively impact vegetation recovery in Tibet (Wu et al., 2012; Zhang and Jin, 2021). Due to the significant decrease in precipitation and extreme catastrophic weather events, the vegetation NDVI in Tibet reached its lowest value within 20 years in 2015. In order to promote vegetation recovery, the Chinese government adopted a series of activities and policies from 2015 to 2016, including construction of protective forest systems, returning farmland to forest, construction of wildlife reserves, and protection of important wetlands, which led to a significant increase in NDVI (Statistical Bureau of Tibet, 2015, 2016).

There are various indicators for the evaluation of human activities, and auxiliary data, such as nighttime light data, have been demonstrated to be effective in facilitating environmental change analysis and vegetation monitoring (Zhang and Seto, 2011; Liu et al., 2014). With the advantages of a wide coverage, high efficiency, and notable visualization, nighttime lights can directly reflect the extent and intensity of human activities and provide the potential to assess socioeconomic development, population migration, carbon emissions, pollution, and environmental monitoring data (Doll et al., 2006; Elvidge et al., 2012; Li et al., 2013). To further determine the causes of NDVI change and vegetation restoration in western Tibet, NPP-VIIRS time-series data were used to analyze the influence of nighttime light data on NDVI changes in this study. The results provided further evidence indicating that human activities were the main factor contributing to vegetation change. As an emerging data source, nighttime light data could be effectively used as an indicator of human activities for vegetation driver identification.

### Limitations and Prospects

The validity of vegetation greenness indicators extracted from different sensors is inconsistent. Remote sensing data such as GIMMS-NDVI and NSMC-NDVI data have been widely used in vegetation monitoring and land change detection (Sha et al., 2013; Wang et al., 2021). However, these data exhibit a low spatial resolution, and the data are no longer updated. MODIS-NDVI data have provided vegetation greenness products since 2001 and exhibit a larger distribution range than that of other data types, which is very effective for timely monitoring of vegetation in large areas (Jepsen et al., 2009). More vegetation indices, such as the enhanced vegetation index (EVI), which can reduce the impact on vegetation canopy background signals and atmospheric effects, exhibit the potential to improve the sensitivity to vegetation in high-biomass areas and have been used for forest parameter mapping and mangrove change detection purposes (Jiang et al., 2020; Samanta et al., 2021). However, in plateau areas with a low vegetation coverage, the NDVI can directly characterize the vegetation coverage. Compared to other indices, the NDVI remains the most commonly used and effective parameter to reflect the change in vegetation greenness (Eastman et al., 2013; Chen et al., 2020).

In addition, the spatial pattern and variation in vegetation greenness may vary drastically with elevation differences (Wang et al., 2021). The topography notably influences the distribution and growth of vegetation, and as one of the regions with the highest average altitude worldwide, the drastic topographic fluctuations and harsh climate in Tibet result in locally obvious differences in the vegetation distribution (Wang et al., 2019, 2022). Spatial autocorrelation can reveal whether and to what extent the attribute characteristics of neighboring elements in geographic space are related and has become a common method for the study of vegetation growth, carbon cycle, heat island effect, and other changes in vegetation ecology and the environment (Li et al., 2021; Yang et al., 2021). Global Moran’s index and local Moran’s index were used to detect the aggregation and local effects of the vegetation NDVI in Tibet, and the results revealed that the NDVI experienced high spatial agglomeration from 2001 to 2020. From 2001 to 2007, global Moran’s index exhibited a fluctuating upward trend, while the NDVI gradually decreased. However, from 2008 to 2020, global Moran’s index greatly fluctuated, demonstrating a downward trend as a whole, reaching the lowest value in 2018 (Figure 4). The main reason is that extreme weather events and disasters frequently occurred in Tibet in 2018, resulting in serious losses of agricultural production and construction facilities. Moreover, there exist obvious differences in the spatial distribution of NDVI accumulation areas in Tibet. Due to a large number of cities and populations, the vegetation distribution in the central region is relatively fragmented, so the aggregation phenomenon is not notable (Zhang et al., 2018). However, the availability of high-resolution remote sensing data is limited by factors such as cloudiness and data computational efficiency, causing difficulties in the exploration of local distribution patterns of vegetation in more detail on long time scales.

The obtained NDVI growth rate was slightly lower than that determined by Chen et al. (2020) because the vegetation restoration area of the Qinghai-Tibet Plateau was mainly located in Sichuan Province and Qinghai Province, which occur in the eastern and northern parts, respectively, of the plateau. These provinces experienced better vegetation recovery due to grazing control, reforestation, and climate change. However, the effect of vegetation recovery was limited in Tibet due to its geographical location and topography, which deserves more attention. In addition, regarding future trends of vegetation, the change trend in most vegetation areas was not notable, and there occurred a risk of degradation, which is similar to the conclusion of Chen et al. (2020).

Global Moran’s indices of the NDVI in Tibet from 2001 to 2020 were extremely significant, which indicated that the aggregation phenomenon was obvious and continuous. However, further validation is necessary, which yields positive implications for the study of the elevation gradient on the distribution and migration of vegetation (Walker et al., 2014). In addition, the vegetation in Tibet at the local scale is fragile, and the control mechanisms of vegetation change are complex. The reasons for the influence of anthropogenic and climatic factors on NDVI trends should be further investigated *via* quantitative analysis.

## Conclusion

In this study, NDVI time-series data from 2001 to 2020 in Tibet were obtained based on MOD13Q1 data retrieved from the Google Earth Engine platform. The coefficient of variation method, Theil–Sen median method with the Mann–Kendall test, and Hurst exponent method were used to identify the spatial and temporal changes and future trends of the vegetation cover characteristics of Tibet, and the main driving forces affecting the changes in vegetation NDVI were analyzed. The main conclusions in this study were as follows: (1) the distribution of NDVI values in Tibet exhibited the spatial characteristics of high values in the southeast and low values in the northwest. The vegetation improvement area accounted for 56.6% of the total study area, the stable unchanged area accounted for 27.5% of the total study area, and the vegetation degradation area accounted for only 15.9% of the total study area. (2) The NDVI changes in Tibetan vegetation over the past 20 years were not very volatile, and the areas with relatively low and moderate fluctuation changes dominated. The areas with high-fluctuation changes were scattered in the west, central, and east, and the areas with low fluctuation changes were mainly distributed in the southeast and northeast. (3) Regarding future change trends, the long time series of the vegetation NDVI in Tibet was generally persistent, and the total area of continuous improvement accounted for 41.2% of the total area of the region, mainly distributed in the northwest and southeast. (4) In regard to the drivers of NDVI changes, overall, climatic factors and population growth did not significantly influence vegetation NDVI changes in Tibet over the last 20 years. However, precipitation and human activities in the west were the main drivers of localized vegetation cover improvement.

## Data Availability Statement

The original contributions presented in the study are included in the article/supplementary material; further inquiries can be directed to the corresponding author.

## Author Contributions

FJ, MD, and YL conceived the study and wrote the paper. HS and FJ implemented the algorithm and conducted formal analysis. All authors contributed to the article and approved the submitted version.

## Funding

This research is supported by the Natural Science Foundation of China (31971578), the Scientific Research Fund of Changsha Science and Technology Bureau (kq2004095), the Scientific Research Fund of Hunan Provincial Education Department (17A225), and the Postgraduate Scientific Research Innovation Project of Hunan Province (CX20210852).

## Conflict of Interest

The authors declare that the research was conducted in the absence of any commercial or financial relationships that could be construed as a potential conflict of interest.

## Publisher’s Note

All claims expressed in this article are solely those of the authors and do not necessarily represent those of their affiliated organizations, or those of the publisher, the editors and the reviewers. Any product that may be evaluated in this article, or claim that may be made by its manufacturer, is not guaranteed or endorsed by the publisher.

## Footnotes

^1^ https://www.resdc.cn/

^2^ https://www.worldpop.org/

^3^ https://eogdata.mines.edu/products/vnl/
